# Development and field validation of a regional, management‐scale habitat model: A koala *Phascolarctos cinereus* case study

**DOI:** 10.1002/ece3.3300

**Published:** 2017-08-11

**Authors:** Bradley Law, Gabriele Caccamo, Paul Roe, Anthony Truskinger, Traecey Brassil, Leroy Gonsalves, Anna McConville, Matthew Stanton

**Affiliations:** ^1^ NSW Department of Industry‐Lands and Forestry Forest Science Unit Parramatta NSW Australia; ^2^ Science and Engineering Faculty Queensland University of Technology Brisbane Qld Australia; ^3^ EchoEcology Sydney NSW Australia; ^4^ Niche Environment and Heritage Sydney NSW Australia

**Keywords:** detectability, ground‐truth, MaxEnt, species distribution models

## Abstract

Species distribution models have great potential to efficiently guide management for threatened species, especially for those that are rare or cryptic. We used MaxEnt to develop a regional‐scale model for the koala *Phascolarctos cinereus* at a resolution (250 m) that could be used to guide management. To ensure the model was fit for purpose, we placed emphasis on validating the model using independently‐collected field data. We reduced substantial spatial clustering of records in coastal urban areas using a 2‐km spatial filter and by modeling separately two subregions separated by the 500‐m elevational contour. A bias file was prepared that accounted for variable survey effort. Frequency of wildfire, soil type, floristics and elevation had the highest relative contribution to the model, while a number of other variables made minor contributions. The model was effective in discriminating different habitat suitability classes when compared with koala records not used in modeling. We validated the MaxEnt model at 65 ground‐truth sites using independent data on koala occupancy (acoustic sampling) and habitat quality (browse tree availability). Koala bellows (*n* = 276) were analyzed in an occupancy modeling framework, while site habitat quality was indexed based on browse trees. Field validation demonstrated a linear increase in koala occupancy with higher modeled habitat suitability at ground‐truth sites. Similarly, a site habitat quality index at ground‐truth sites was correlated positively with modeled habitat suitability. The MaxEnt model provided a better fit to estimated koala occupancy than the site‐based habitat quality index, probably because many variables were considered simultaneously by the model rather than just browse species. The positive relationship of the model with both site occupancy and habitat quality indicates that the model is fit for application at relevant management scales. Field‐validated models of similar resolution would assist in guiding management of conservation‐dependent species.

## 1 INTRODUCTION

Distribution modeling provides effective tools for spatially‐explicit mapping of relationships between species occurrences and biophysical variables over broad extents (e.g., Yost, Petersen, Gregg, & Miller, 2008). Predictive models of species distributions have become increasingly important conservation tools for guiding and informing on‐ground management, especially for threatened species that are difficult to reliably survey (Dickson et al., 2014; Liu, White, Newell, & Griffioen, 2013). However, models relying on “presence only” records are influenced by detection issues and sampling bias, and there is a need to ensure they are fit for purpose (e.g., predicting relative site suitability; Guillera‐Arroita et al., 2015). Such limitations reinforce the need for independent field validation of models as a key step when implementing species distribution models for management purposes (Hirzel, Le Lay, Helfer, Randin, & Guisan, 2006; Latif, Saab, Mellen‐Mclean, & Dudley, 2015; Loehle, Irwin, Manly, & Merrill, 2015). Field validation can assess the accuracy of modelled predictions and their potential benefit to conservation planning management, although frequently this step is not undertaken or possible (Araújo, Pearson, Thuiller, & Erhard, 2005).

One impediment with field validation is confident identification of varying habitat quality and establishing whether it is occupied, and particularly, to avoid false absences by accounting for imperfect detection when estimating occupancy (MacKenzie et al., 2002; Wintle, Kavanagh, McCarthy, & Burgman, 2005). Both the fit and relationship of predicted occupancy values against a model's output allow field validation based on ground‐truth data. As an alternative to occupancy, given that not all habitats of rare species are likely to be occupied, habitat quality can be assessed independently based on the known habitat or dietary preferences of the focal species (e.g., browse species). This approach is limited by available knowledge on the importance of different browse species and how this varies across a species range or in association with co‐occurring browse species, soil type, moisture, disturbance, etc.

The koala (*Phascolarctos cinereus*) is an iconic arboreal marsupial occurring in eucalypt forests and woodlands across eastern Australia (Martin, Handasyde, & Krockenburger, 2008). The species is in decline (e.g., Adams‐Hosking, Grantham, Rhodes, McAlpine, & Moss, 2011) and is listed as an endangered species. A recent Australian Senate inquiry (Commonwealth of Australia, 2011) recommended the implementation of habitat mapping to assist in the management of the koala, highlighting the need for reliable distribution models for this species. Regional, coarse resolution models have been produced for koalas that map likelihood of records (Predavec et al., 2015) or that predict suitability based on a variety of data‐layers (Santika, McAlpine, Lunney, Wilson, & Rhodes, 2014; Sequeira, Roetman, Daniels, Baker, & Bradshaw, 2014). In comparison, fine‐resolution maps (i.e., 25 m) of koala distribution based on associations between fecal pellet counts and floristic associations have been restricted to local scales (e.g., Callaghan et al., 2011; Lunney, Phillips, Callaghan, & Coburn, 1998). Regional models prepared at a scale suitable for management maximize the usefulness of koala habitat maps for land managers, especially where a complex mosaic of habitat quality in local areas can be expected. Finer resolution models would also allow management actions to target the most appropriate areas for conservation (Razgour, Hanmer, & Jones, 2011), such as guiding tree retention levels in high‐quality koala habitat in timber production forests (Predavec et al., 2015).

Being an obligate folivore, koalas are typically associated with particular species of *Eucalyptus* that provide palatable foliage (DECC 2008; Phillips, Callaghan, & Thompson, 2000), although browse species preference may vary because of differences in site productivity or because the availability of more desirable tree species varies (Crowther, McAlpine, Lunney, Shannon, & Bryant, 2009; Moore, Lawler, Wallis, Beale, & Foley, 2010; Phillips & Callaghan, 2000). In addition to availability of preferred *Eucalyptus* species, koala habitat is likely to be influenced by other factors such as habitat loss and fragmentation (e.g., McAlpine et al., 2006; Rhodes et al., 2006). The combined effect of environmental factors (e.g., topography, climate) and disturbances (e.g., fire) results in a spatially complex array of tree species within Australian eucalypt forests (Coops & Catling, 2000) and, consequently, a mosaic of suitable and less suitable conditions for koalas. Coarse resolution koala habitat models (e.g., 5 km) may not adequately capture the level of spatial complexity needed to provide suitable information for local‐scale management. This can occur where there is a mismatch between the resolution of the model and the key environmental features determining habitat quality (Guerrero, Mcallister, Corcoran, & Wilson, 2013; Hermoso & Kennard, 2012), leading to limited implementation of the model for management purposes (Tulloch et al., 2016).

In this study, we modeled the potential habitat of koalas at a resolution suitable for land management (i.e., 250 m) across northeastern NSW using the Maximum Entropy Approach (MaxEnt, Phillips, Anderson, & Schapire, 2006). MaxEnt is a powerful machine learning technique that models “presence only” records (Elith et al., 2011) to produce environmental niche and species distribution maps (hereafter habitat suitability models). Our aim was to develop and validate a predictive habitat suitability model that would be useful for managing the species in the context of forest management, especially timber harvesting. To achieve this, our objective was to ensure the model's resolution was fine enough to map habitat suitability for the species at a forest subcompartment scale (250‐m grid cell). Rather than rely on cross‐validation approaches that have problems related to data dependence (Roberts et al. 2017), we field validated our koala model by relating modeled habitat suitability to two different, although complementary, independent datasets at ground‐truth sites. The first dataset of koala occupancy was estimated using acoustic sensors set over a seven night period, allowing detectability to be accounted for. The second was an index of koala habitat quality based on browse species availability. For model validation purposes, we predicted an increase in koala occupancy and the habitat quality index with modeled habitat suitability across all ground‐truth sites.

## 2 METHODS

### 2.1 Study area and koala occurrence records

**Figure 1:**
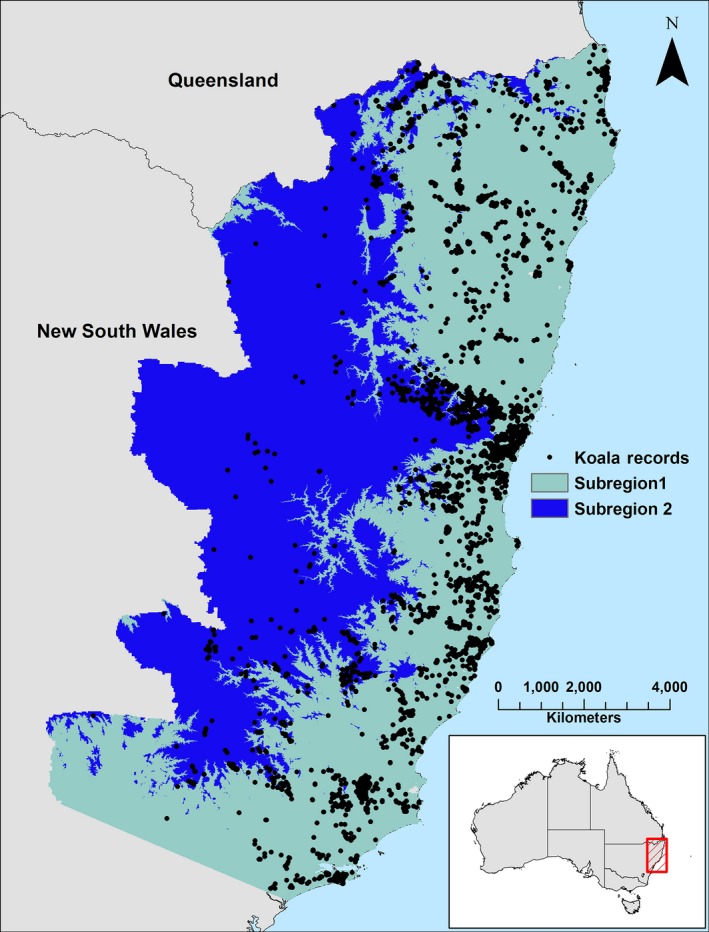
Map of northeast NSW with the locations of 5,558 koala records within the two subregions

The analysis focused on northeastern New South Wales. The study area (~8.5 million ha) consisted of two subregions: subregion 1 (areas below 500 m above sea level (ASL)) and subregion 2 (areas above 500 m ASL) (Fig. 1). This subdivision was chosen because it was considered likely that different drivers of koala habitat operated in coastal areas compared to uplands (McAlpine et al., 2008). We acquired reliable locations (*n* = 7,997, <100‐m accuracy) where koalas have been recorded (1990–2015) from the New South Wales National Parks and Wildlife Service Wildlife Atlas. Records dating back to 1990 were used because extensive forest surveys were undertaken for the koala in the early 1990s that have not been repeated, and inclusion of these records better represents koala distribution than relying on only more recent records. All records located within cleared areas were removed reducing the number of suitable records to 5,558 (4,238 in Subregion 1 and 1,320 in Subregion 2). In order to reduce spatial aggregation in our records (e.g., Fourcade, Engler, Rödder, & Secondi, 2014; Kramer‐Schadt et al., 2013), we randomly selected koala occurrences that were separated by a minimum distance of 2 km. We replicated this filtering process five times and generated five random sets of records for each subregion. The number of records in the five sets ranged from 1,078 to 1,090. Records (*n* = 3,116) that were not included in the five filtered sets were retained and used for model evaluation (see Section 2.4) prior to field validation.

### 2.2 Environmental variables

We trialed 30 gridded (250 m) environmental variables for their potential influence on koala habitat suitability (Table 1). These were selected from published relationships between koalas and their environments, such as the influence of vegetation, browse trees, topography, fire, climate, and primary productivity on the distribution of koalas (Lunney, Gresser, O'Neill, Matthews, & Rhodes, 2007; Van Dyck and Strahan, 2008; Moore et al., 2010; Sequeira et al., 2014; Briscoe, Kearney, Taylor, & Wintle, 2016). Variables included both biotic (e.g., floristic composition) and abiotic (e.g., climate, soil) factors and were produced at a 250‐meter spatial resolution (i.e., pixel size = 250 m).

**Table 1:** List of the 30 environmental variables trialed in the MaxEnt predictive modeling

Variable name	Variable description	Variable type
**Climatic variables**		
Bio01	Annual mean temperature (°C)	Continuous
Bio08	Mean temperature of wettest quarter (°C)	Continuous
Bio09	Mean temperature of driest quarter (°C)	Continuous
Bio10	Mean temperature of warmest quarter (°C)	Continuous
Bio11	Mean temperature of coldest quarter (°C)	Continuous
Bio12	Annual precipitation (mm)	Continuous
Bio14	Precipitation of driest period (mm)	Continuous
Bio17	Precipitation of driest quarter (mm)	Continuous
Bio20	Annual mean radiation (Mj/m^2^/day)	Continuous
Bio28	Annual mean moisture index	Continuous
**Vegetation variables**		
Biomass	Above ground biomass (Mg Ha^−1^)	Continuous
Cra	CRAFTI floristic groups: Class 1: Primary browse species Class 2: Secondary browse species Class 3: Tertiary browse species Class 4: Unsuitable habitat	Categorical
Cra%	Percentage cover of primary and secondary CRAFTI‐based browse species	
Fpc	Foliage projective cover (%)	Continuous
NDVI_au	Normalized difference vegetation index in autumn	Continuous
NDVI_sp	Normalized difference vegetation index in spring	Continuous
NDVI_su	Normalized difference vegetation index in summer	Continuous
NDVI_wi	Normalized difference vegetation index in winter	Continuous
NPP	Net primary productivity (kg C/m^2^)	Continuous
**Disturbance variables**		
Fire	Wildfire frequency (1970–2015): Class 0: areas that never burned and that are considered not flammable (e.g., rainforests) Class 1: areas that never burned Class 2: areas that burned 1–3 times Class 3: areas that burned more than 3 times	Categorical
Sea	Density of sealed roads (m of road per km^2^)	Continuous
**Topographic variables**		
DEM	Digital elevation model (m)	Continuous
Slo	Slope (degree)	Continuous
Top	Topographic position index	Continuous
Tor	Topographic roughness (m)	Continuous
**Soil variables**		
Asc	Australian soil classification: Class1 = Anthroposols; Class2 = Calcarosols; Class3 = Chromosols; Class4 = Dermosols; Class5 = Ferrosols; Class6 = Hydrosols; Class7 = Kandosols; Class8 = Kurosols; Class9 = Organosols; Class10 = Podosols; Class11 = Rudosols; Class 12 = Sodosols; Class13 = Tenosols; Class14 = Vertosols	Categorical
Awc	Available water capacity (%)	Continuous
Dep	Soil depth (m)	Continuous
Oc	Organic carbon (%)	Continuous
Tp	Total phosphorus (%)	Continuous

Four broad floristic categories (1 = primary browse species, 2 = secondary browse species, 3 = tertiary browse species, and 4 = unsuitable habitat) were derived from available floristic maps (i.e., Comprehensive Regional Assessment Aerial Photographic Interpretation, CRAFTI; NSW National Parks and Wildlife Service, 2001a,b). Categorization was based on prevalence of tree species in each forest type and the importance of tree species to koalas as listed in the NSW Koala Recovery Plan (Department of Environment and Climate Change, 2008) and supplemented by expert opinion. Additionally, we calculated the percentage cover of Class 1 and Class 2 (CRAFTI) combined within a 1‐km radius of each pixel to account for the coverage of primary and secondary browse species at a broader scale. Four topography‐related variables were used (Table 1) together with the density of sealed roads (m of road per km^2^) to account for anthropogenic disturbance.

Soil types were derived from the National Soil Data provided by the Australian Collaborative Land Evaluation Program ACLEP (http://www.clw.csiro.au/aclep/, last accessed February 2017). Additionally, we acquired soil depth (m), organic carbon (%), total phosphorus (%), and available water capacity (%) from The Soil and Landscape Grid of Australia (http://www.clw.csiro.au/aclep/soilandlandscapegrid/ProductDetails-SoilAttributes.html, accessed April 2016) to characterize soil fertility, which is thought to be an important influence on browse quality (Moore et al., 2010). We also derived site greenness from remote‐sensing variables, calculating normalized difference vegetation index (NDVI, Rouse, Haas, Schell, & Deering, 1974) values using MODIS MOD13Q1 granules acquired in January, April, July, and October from 2000 to 2015. For each month, all NDVI data were averaged to provide spectral values in the central month of summer (NDVI_su), autumn (NDVI_au), winter (NDVI_wi), and spring (NDVI_sp). Three additional vegetation‐related variables were included in the analysis: (1) above ground biomass (Mg Ha^−1^) was acquired from NSW Office of Environment and Heritage (ALOS Woody biomass, Lucas et al., 2010), (2) foliage projective cover (%) was acquired from NSW Office of Environment and Heritage (http://www.environment.nsw.gov.au/research/AncillaryVegetationProductsDataInventory.htm, last accessed February 2017), and (3) net primary productivity (NPP, kg C/m^2^) was extracted from MODIS data (MOD17A3). MOD17A3 annual NPP was averaged from 2000 to 2015 to calculate mean annual NPP. A number of bioclimatic factors were investigated for their potential influence on the distribution of koalas. Bioclim (Houlder, Hutchinson, Nix, & McMahon, 2009) was used to produce 10 bioclimatic parameters based on long‐term meteorological data and a digital elevation model (DEM). Finally, we used wildfire history data (1970–2015) acquired from NSW Rural Fire Service for the potential influence of this disturbance on koala habitat suitability. A fire frequency map was calculated by classifying the data into four categories (Table 1).

An essential step in habitat modeling is to avoid overfitting using too many variables, especially those that are highly intercorrelated. To minimize multicollinearity, the number of continuous variables was reduced by eliminating highly correlated (*R* > 0.75) predictors and retaining the variable with the most interpretable biological response (Kramer‐Schadt et al., 2013).

### 2.3 Bias file

Many koala records were collected via community surveys and so are biased to locations frequently visited by people, especially along the coast where urban centers are located. To reduce the effect of this bias, a *bias* file was created to account for sampling effort of records held in a public database. Following Predavec et al. (2015), we estimated sampling intensity using the aggregation of occurrences for arboreal mammal species (taxonomic groups: Petauridae, Phalangeridae, Phascolarctidae, and Pseudocheiridae) that are likely to reflect detectability of the koala. A gaussian kernel density map of koala and arboreal mammal occurrences was generated and rescaled to 1–30 (Fourcade et al., 2014). Values in the resulting map were higher in densely sampled areas indicating higher sampling effort (e.g., near urban centers).

### 2.4 MaxEnt modeling

Koala habitat suitability in subregion 1 and subregion 2 was modeled separately using MaxEnt. For each run, hinge feature type was used (after Phillips & Dudik, 2008), and maximum number of iteration, convergence threshold, regularization multiplier, maximum number of background points were set to 1,000, 10^−5^, 2, and 10,000, respectively. Optimal regularization was selected by comparing alternatives in ENMTOOLS. We modeled the five spatially filtered sample sets of koala records separately by running 20 replicates for each set (i.e., random partitions of 75% training and 25% testing data) and retaining the mean predicted habitat suitability. Finally, we averaged the mean predicted habitat suitability for each of the five sample sets to generate the final koala habitat suitability map. Each pixel in MaxEnt logistic output is assigned with a value ranging from 0 to 1 representing the relative occurrence rate of suitable environmental conditions for the target species (habitat suitability). We used the receiver operating characteristic (ROC) curve on test data to evaluate the model's performance. The area under the ROC curve (AUC) provides a single indicator of model performance (Phillips et al., 2006), with AUC > 0.7 indicating good discriminatory power (Hosmer & Lemeshow, 1989).

We analyzed the relationship between koala records (*n* = 3,116) that were not used in the MaxEnt analysis (see section “*Study area and species occurrence records*”) and the predictive habitat suitability model output. These records were neither filtered nor adjusted based on survey effort. Finally, we analyzed the response curves of the predictor variables to assess their influence on the prediction. Response curves show how predicted suitability of a model built using only one variable changes as it is varied.

### 2.5 Validation of model using independent field data

#### 2.5.1 Site selection

To ground‐truth the koala MaxEnt Model, we established 65 sites in different land tenures (including timber production landscapes) across the study area from the coast to over 1,000 m in altitude (Appendix S1). Sites with a recent history of logging or fire (<5 years) were avoided as recent disturbance would influence model validation if koalas were absent. Allocation of sites was stratified using four habitat quality classes (very high, high, moderate, low) derived from a preliminary version of the koala habitat suitability model (Law et al., unpubl.). Ground‐truth sites were evenly spread between lower slopes (*n* = 28) and upper slopes (*n* = 32) with a small sample from midslopes (*n* = 5). Areas with unsuitable habitat for koalas, such as heath or swamp, were not included as ground‐truth sites.

#### 2.5.2 Koala occupancy

Koala males emit loud bellows during the breeding season (Ellis et al., 2011) allowing this behavior to be used for estimating koala occupancy. At each ground‐truth site, we deployed one SongMeter (SM2 – Wildlife Acoustics) to record koala bellows. SongMeters were programmed to record from one hour before sunset until sunrise for seven consecutive nights. Two of the 65 SongMeters failed to record data, leaving us with occupancy data for 63 sites (441 sample nights). The distance at which koala calls can be detected is likely to vary with environmental conditions, but bellows are considered to be detectable by SongMeters up to at least 100 m (W. Ellis personal communication). All SongMeter sampling was undertaken in the koala mating season across three trips in 2015; one trip in October/November, one in late November, and one in December.

#### 2.5.3 Analysis of koala calls

Recordings were scanned by acoustic software and a koala recogniser (Towsey, Planitz, Nantes, Wimmer, & Roe, 2012). Recordings matched by the koala recogniser were checked for false positives by manually visualizing spectrograms of the audio and listening to recordings, while random checks were carried out for false negatives. A single koala call was made up of multiple event triggers. We defined a koala call as sequential event triggers that were <60 s apart. The number of koala calls was manually tallied to give the total number of koala calls per site per night.

#### 2.5.4 Occupancy analysis and validation method

We used an occupancy modeling framework to account for imperfect detection of koala bellows at sites and estimate probability of site occupancy (MacKenzie et al., 2002). We used data from seven consecutive nights of sampling to estimate the probability of detection and used this to calculate probability of occupancy in PRESENCE version 10.5 (Hines, 2006). For the validation of the MaxEnt model, probability of occupancy per site was estimated by incorporating the MaxEnt modeled habitat suitability for each ground‐truth site as a covariate (predictor) in a regression relationship. The fit of this relationship against koala occupancy was compared, via model selection procedures, with other potentially important site covariates. Competing models were ranked using Akaike Information Criterion (AIC), which measures the trade‐off between model complexity (number of parameters) and precision (fit) of the models. The difference between each model's AIC value and the best‐fitting model was calculated, with models of delta AIC < 2 from the best model considered to have substantial support.

Modeling followed a multistaged process.

1. We identified the importance of possible covariates for koala detectability to improve the accuracy of occupancy estimates. Daily rainfall (p(rainfall)), month of sampling trip (p(trip)), and topographic position (p(topo)) were compared against a null model with constant detectability (p(.)).
2. Using results for detectability (Step 1), we compared the strength of the relationship between koala occupancy, the MaxEnt modeled habitat suitability, the site habitat quality index (see 2.5.5), and a null model of constant site occupancy (Psi(.)).We also included a selection of other potential predictors of koala habitat (NPP, topographic position, elevation, and wildfire frequency) that were extracted for the 250‐m pixel for each of the ground‐truth sites.

#### 2.5.5 Site habitat quality

The second approach for field validation was a site‐based assessment of habitat potential for koalas. To quantitatively assess browse tree availability at each site, a 200‐m transect was established and at every 20‐m interval, the Point‐Quarter technique (Pollard, 1971) was employed to measure the distance to the nearest tree (>20‐cm diameter at breast height, dbh) in each quadrant. Each tree was identified to species where possible, and its diameter was measured and height estimated. This resulted in data on 40 trees from 10 points along each transect. The Point‐Quarter technique was then used to estimate stem density and when multiplied by the % occurrence of different species and their mean diameter, we were able to calculate the basal area (to account for tree size) for the different species measured. An index of habitat quality for koalas at each ground‐truth site was calculated based on browse tree basal area and diversity (Appendix S1).

## 3 RESULTS

### 3.1 MaxEnt modeling

**Figure 2:**
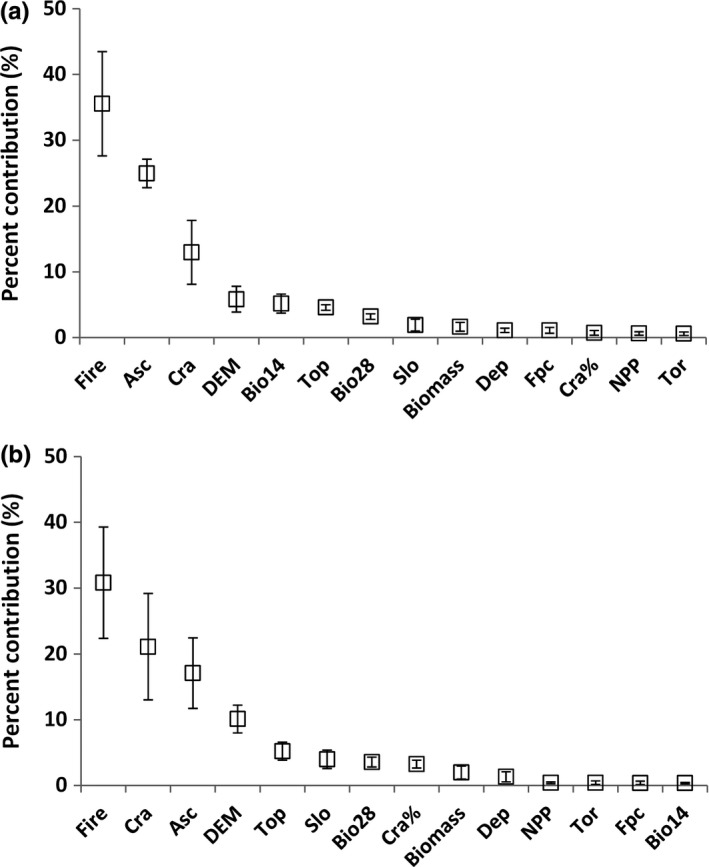
Percent contribution of the 14 predictor variables in (a) subregion 1 and (b) subregion 2. See Table 1 for environmental variables description

A large number of continuous variables were highly correlated (*R* > 0.75) and were therefore excluded from MaxEnt modeling. Some of the continuous variables initially retained (i.e., water‐holding capacity, organic carbon and phosphorus, and sealed roads; Table 1) were also discarded after exploratory analysis showed their response curves lacked realism and ecological sense. Therefore, the models for subregion 1 and subregion 2 were built on a total of 14 predictors: three categorical variables (soil type (Asc), vegetation type (Cra), and wildfire frequency (Fire; Table 1)) and 11 continuous variables (climatic variables (Bio14, Bio28), vegetation quantity (Biomass, Fpc, local landscape extent of preferred vegetation types (Cra%), elevation (DEM), soil depth (Dep), site productivity (NPP), and topography (Slo, Top and Tor; Table 1)). AUC ranged from 0.736 to 0.752 (*n* = 5, mean ± *SE *= 0.741 ± 0.006) for subregion 1 and from 0.786 to 0.801 (*n* = 5, mean ± *SE* = 0.796 ± 0.006) for subregion 2. For both subregions, Asc, Cra, DEM, and Fire provided the greatest contribution to the model (Fig. 2).

**Figure 3:**
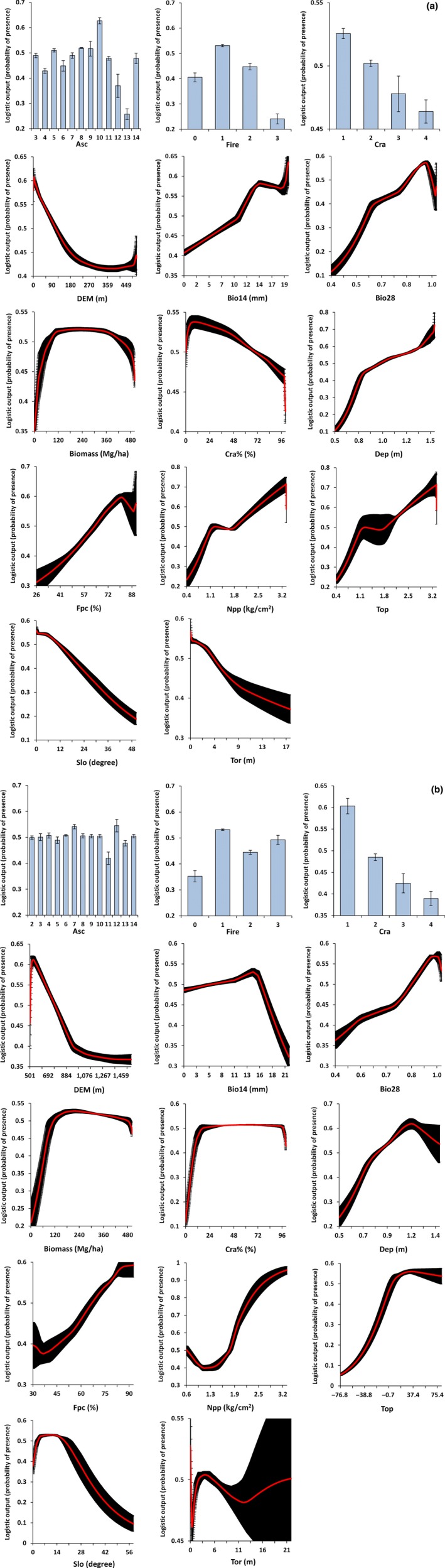
Response curves from MaxEnt modeling of koala records for (a) subregion 1 and (b) subregion 2. See Table 1 for environmental variables description

The response curves of Asc, Cra, Fire, and DEM (Fig. 3a,b) showed some differences between the two subregions. Predicted suitability of Asc was higher for Class 10 (Podosols) in subregion 1 and Class 12 (Sodosols) in subregion 2, while Class 13 (Tenosols) and Class 11 (Rudosols) showed the lowest probability values for subregion 1 and subregion 2, respectively. Predicted suitability of Cra was higher for Class 1 and decreased gradually from Class 2 to Class 4 in both subregions. Predicted suitability of Fire showed similar values for Class 0, Class 1, and Class 2 (~36%, ~53%, and ~44%, respectively) in both subregions. However, Class 3 (high frequency of wildfire) showed a markedly higher predicted suitability in subregion 2 (~49%) when compared to subregion 1 (~24%). The response curve of DEM showed a similar pattern in both subregions as predicted suitability decreased for higher values. High predicted suitability <100 m and between 500 and 600 m elevation, reflect a concentration of koala records at those elevations.

**Figure 4:**
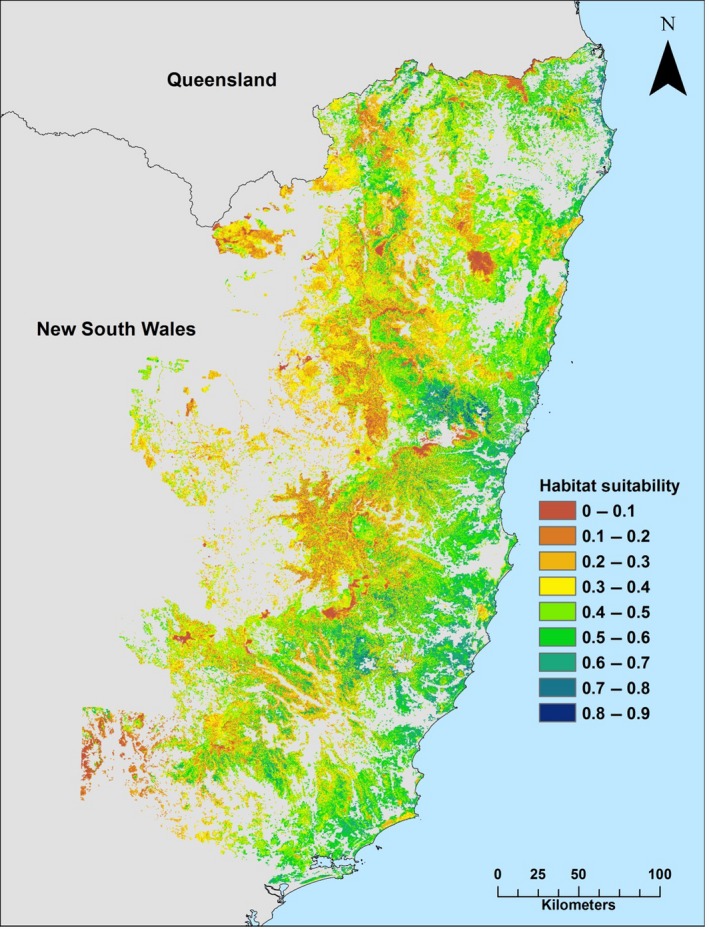
Koala habitat suitability map from MaxEnt modeling in northern NSW. Nine categories of habitat suitability are shown. Areas cleared of native vegetation (i.e., gray) were not modeled

**Figure 5:**
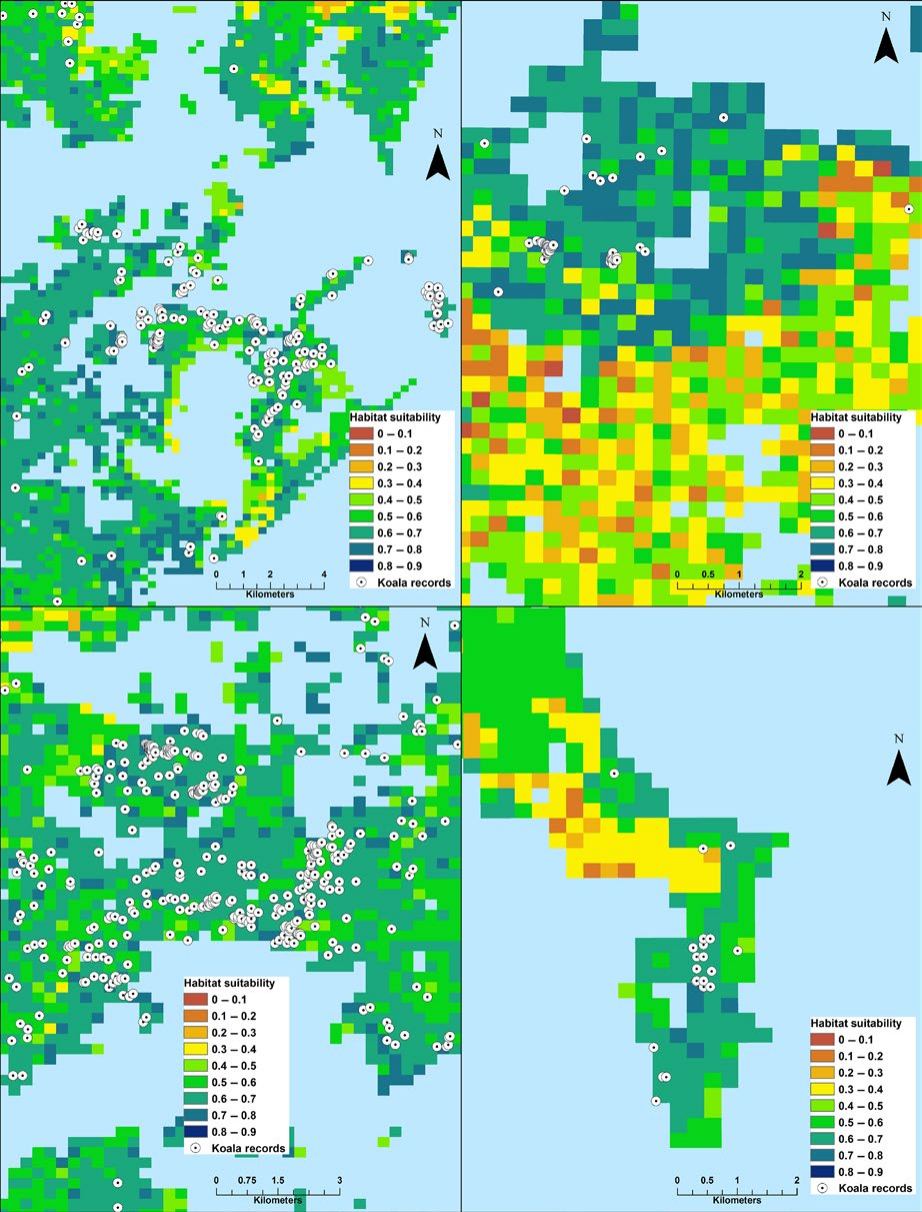
Examples of four areas of koala habitat suitability characterized by high record density

Habitat suitability values ranged from 0 to 0.88 (mean ± *SE* = 0.39 ± 0.15) and were classified into nine categories corresponding to 0.1 increments (Fig. 4). Most of the areas characterized by high frequency of koala records (Fig. 5) were correctly modeled and assigned with high or very high suitability classes. Koala records less frequently fell in areas modeled as moderate suitability and rarely in low suitability habitat.

**Figure 6:**
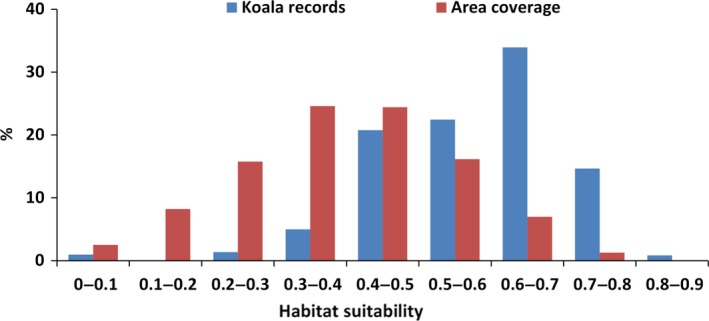
Distribution of area coverage (%) and koala records (%) within nine ranges of habitat suitability classes. Koala records are those not used in model development

We analyzed the frequency of the 3,116 koala records that were excluded from MaxEnt analysis within the nine modeled suitability classes. The frequency of the nine classes was unimodal with >75% of the study area recording habitat suitability values ≤0.5 (Fig. 6). The distribution was unequal across the classes, and ~50% of the records were located in areas with habitat suitability >0.6, representing ~8% of the study area. The highest frequency of records (~34%) was recorded between 0.6 and 0.7. Only ~7% of the records were located in areas with suitability <0.4, yet this constituted ~51% of the study area.

### 3.2 Field validation using koala occupancy

A total of 276 koala bellows were recorded on 46 out of 441 nights of sampling at 29% of sites. A high number (>20) of koala calls were recorded at the following sites: Braemar State Forest, Wild Cattle Creek State Forest, Chichester State Forest, Pine Creek State Forest, and Yabbra State Forest.

Modeling of detection probability indicated that constant detection was the best supported model (Table 2), with a low probability of detection per night of 0.32. However, varying detectability by trip fell within two AIC points of the top model and so was also supported (although with half the AIC weight). Koala detectability declined slightly from 0.43 in October/November to 0.36 in late November to 0.30 in December. Neither daily rainfall nor a topographic position index influenced detectability.

**Table 2:** Model selection results for ground‐truth sites comparing the null model (constant detection) with alternative models that allow koala detectability to covary with daily rainfall, month of survey (trip), and topographic position

Model	AIC	Delta AIC	AIC weight	Model likelihood	No. parameters	−2*Log likelihood
psi(.),p(.)	238.74	0.00	0.6657	1.0000	2	234.74
psi(.),p(trip)	240.22	1.48	0.3176	0.4771	2	236.22
psi(.),p(rainfall)	246.12	7.38	0.0166	0.0250	2	242.12
psi(.),p(topo)	253.2	14.46	0.005	0.0007	2	249.2

**Figure 7:**
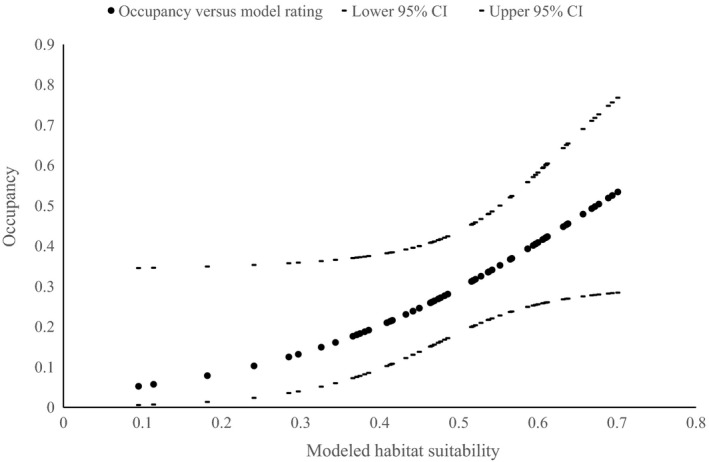
Model validation results from 63 ground‐truth sites. The graph shows the relationship between the fitted probability of koala occupancy (after accounting for detectability) against the MaxEnt modeled habitat suitability at a 250‐m pixel scale. Values are the mean fitted values ± 95% confidence intervals (i.e., predicted from the MaxEnt model)

Modeling of occupancy per site against the MaxEnt modeled habitat suitability calculated at the 250‐m pixel scale surrounding each ground‐truthed site revealed a near linear relationship between fitted values of site occupancy and the MaxEnt model output (Fig. 7). In other words, an increase in model output was correlated positively with koala occupancy (*df* = 62, *r* = 0.681, *p* < 0.001). The data were considered to be a good fit to this model as assessed by the Pearson chi‐squared statistic (chi‐square = 338.349, *p *=* *0.10, chat = 1.5781). A similar pattern was evident when the two subregions were validated separately.

### 3.3 Field validation using the site habitat quality index

**Figure 8:**
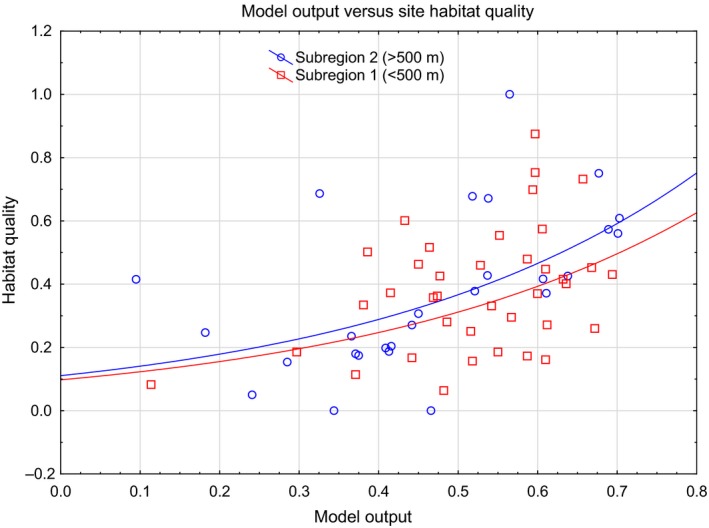
Model validation using the relationships between a habitat quality index based on browse tree availability and diversity with each MaxEnt model output for 65 ground‐truth sites. Ground‐truth sites for each of two subregions are shown separately

The MaxEnt model was significantly correlated with site quality for koalas at ground‐truth sites as represented by the site habitat quality index derived from browse tree availability. The site index increased positively with the MaxEnt output in both subregions (Subregion 1: *r* ^2^ = 0.29; *p* = 0.0039; Subregion 2: *r* ^2^ = 0.15, *p* = 0.017) (Fig. 8). The relationship was weaker, although still significant, for the high elevation subregion 2, where there was more scatter and fewer ground‐truth sites. Some of the variability in the relationship can be attributed to a group of rainforest sites that are potentially overpredicted by the model (Fig. 8). These were typically small patches in close proximity to eucalypt forest.

### 3.4 Koala occupancy versus MaxEnt model, habitat quality index, and other predictors

The MaxEnt model was a better predictor of koala occupancy than the site habitat quality index that was based solely on browse trees (Table 3). When assessed individually, other site attributes including NPP, topographic position, elevation, and the frequency of wildfires were also poorer predictors of koala occupancy than the MaxEnt model.

**Table 3:** Model selection results comparing the null model (constant occupancy) with models allowing koala occupancy at ground‐truth sites to covary with the 250‐m scale MaxEnt model output (psi 250 m), habitat quality index (psi habitat quality), and other site attributes calculated for each ground‐truth site. Detectability was held constant

Model	AIC	Delta AIC	AIC weight	Model likelihood	No. parameters	−2*Log likelihood
psi(250 m),p(.)	236.25	0.00	0.5152	1.0000	3	230.25
psi(.),p(.)	238.74	2.49	0.1483	0.2879	2	234.74
psi(npp),p(.)	239.76	3.51	0.0891	0.1729	3	233.76
psi(topo),p(.)	240.07	3.82	0.0763	0.1481	3	234.07
psi(elevation),p(.)	240.55	4.3	0.06	0.1165	3	234.55
psi(fire),p(.)	240.69	4.44	0.056	0.1086	3	234.69
psi(habitat quality),p(.)	240.07	3.82	0.0551	0.1481	3	234.07

## 4 DISCUSSION

We demonstrate the value of field‐validated species distribution modeling for conservation‐dependent species, using the iconic, koala, as a case study. Our spatially‐explicit model of koala habitat at a 250 m resolution is suitable for guiding management of koalas in timber production forests and other land‐uses or managing wildfire. For example, forestry compartments (~200 ha) form the basic planning unit for timber harvesting and a model of habitat suitability at the resolution of a subcompartment scale (~6 ha) would allow efficient targeting of management actions (e.g., browse tree retention) to areas modeled as high suitability and avoid areas modeled as low suitability. However, we also urge that such applications are coupled with an adaptive management process so that the effectiveness of management for target species is carefully monitored.

Our model corrected for the high spatial bias in the distribution of koala records and was evaluated statistically as a good fit to existing koala records. Most importantly, independent ground‐truthing data demonstrated that the model was reliable for predicting both potential habitat quality and koala occupancy. Previous large‐scale maps of koala likelihood have been produced at a much coarser resolution (e.g., 10 km) (Predavec et al., 2015), which may be more difficult for managers to apply at local scales, although such maps could work in concert with habitat suitability maps at finer scales. About 1.66 million ha were predicted to support moderate‐ to high‐quality habitat for koalas in northeast NSW. Such a large area could support a substantial koala population given relatively high occupancy levels recorded during ground‐truthing.

### 4.1 Model drivers

Our model identified areas of high habitat suitability for koala as those with low wildfire frequency over the past 45 years. High‐intensity fires burn the canopy and can cause death or injury to koalas and a temporary reduction in the availability of foraging habitat (Lunney et al., 2007). The north coast region of NSW has had the second highest number of fires of any region in NSW (behind Sydney) (Bryant, 2008), although it is unknown whether fire severity is higher in that region. Fire severity affected the occurrence of arboreal mammals in Victorian forests, with gullies and unburnt forest serving as refuges (Chia et al., 2015). One implication of the importance of wildfires is that while an area may support a suitable suite of conditions for koalas, such habitat may be unoccupied due to mortality from fire. Other historical factors or current threats including fragmentation by urbanisation, predation by dogs, or extreme climatic events (e.g., drought and heat waves—Lunney, Stalenberg, Santika, & Rhodes, 2014) may similarly reduce koala occupation levels in suitable habitat. The effect of logging on habitat suitability for koalas warrants further investigation (Kavanagh, Debus, Tweedie, & Webster, 1995; Smith, 2004), although in our study ground‐truth sites with many bellows all had a long history of logging.

Koalas also had a lower likelihood of occurrence on Tenosol and Rudosol soils. Tenosols are generally sandy with very low productivity and chemical fertility, poor structure, and low water‐holding capacity (Northcote et al., 1960–1968). Rudosols tend to be shallow with little soil development and are often gravely or rocky. Podosols and Sodosols were predicted to have higher suitability for koalas, and these soils have high organic matter and occur either in coastal areas (Podosols) or in areas with poor drainage (Sodosols), yet both are considered to be relatively infertile. As an example, many koala records in the Port Stephens area occurred on Podosol soils, which are likely to be associated with Swamp Mahogany, *Eucalyptus robusta*, a preferred browse species in this and other coastal areas (Phillips et al., 2000). A direct measure of soil fertility was not supported during model building, possibly because better quality soils have been selectively cleared for agriculture and these were masked from our modeling process.

Floristic composition was the third important variable contributing to the koala model. Habitat suitability was higher on areas mapped with primary browse species, including red gum species (e.g., *Eucalyptus tereticornis*), Tallowwood (*E. microcorys*) and Swamp Mahogany (*E. robusta*) and lower in areas typed as unsuitable habitat (e.g., *Banksia* heath, rainforest with no eucalypt emergents). The two intermediate floristic classes for koala suitability had less discriminating ability, probably because many of the constituent forest types are broad classifications of forest that support varying frequencies of browse species. For example, Blackbutt *Eucalyptus pilularis* and Spotted Gum *Corymbia variegata* types are widespread and not considered highly suitable for koalas (e.g., Phillips et al., 2000), although the frequency of Tallowwood and Grey Gum *Eucalyptus punctata*, two primary browse species, is highly variable in these forest types.

Elevation was the fourth important variable in the koala model. Habitat suitability was predicted to be higher at low elevations in subregion 1, but it was also predicted to be high at 500–600 m in subregion 2. Elevations of 200–500 m and >800 m were predicted to have lower suitability, although with other factors modifying this effect. This pattern of a low and midelevation peak for koalas is related to the extensive number of records in coastal areas and in the Dorrigo plateau and adjacent to Comboyne plateau. An association with low elevations has long been known (e.g., Kavanagh et al., 1995; Phillips et al., 2000; Smith, 2004); however, high habitat suitability at midelevation and even some high elevations (e.g., Nowendoc) appears to be less widely appreciated (but see Krockenberger, 1993; Kavanagh & Stanton, 1995; Braithwaite, 1996). Notably, the New England Tablelands (and the north coast NSW) are predicted to provide climate refugia for koalas under climate change scenarios (Briscoe et al., 2016).

Other variables made minor contributions to the koala model, such as a greater likelihood of koalas on flatter terrain and where soil depth, primary productivity, biomass, and Fpc were higher. The contributions of variables differed somewhat between subregions, such as a greater importance in subregion 1 than subregion 2 for precipitation in the driest quarter. A landscape effect of the surrounding area of preferred forest types had less influence in subregion 1 where there were many koala records in fragmented forest. Although such variables made minor contributions to the model over the entire region, their omission resulted in localized, substantial changes to the model output justifying their inclusion.

### 4.2 Model validation and performance

Our process of validation gave emphasis to field validation over cross‐validation and the resulting AUC score, although the two approaches produced consistent results. The AUC score indicated the model had good discriminatory ability and this was confirmed by field validation at ground‐truth sites. Koala occupancy (adjusted for detectability) at ground‐truth sites increased in a near linear pattern as MaxEnt modeled habitat suitability output values increased. The MaxEnt model output at a 250‐m scale was a stronger performer than larger spatial scales (authors unpubl. data), indicating that more extensive areas of higher habitat suitability than a 250‐m pixel were not better predictors of koala occupancy. This is consistent with the fact that the landscape variable, percentage cover of primary and secondary CRAFTI forest types, was a minor contributor to the MaxEnt model. Such a result contrasts with local studies in fragmented rural areas that have identified the importance of landscape context, patch size, fragmentation, and connectivity (McAlpine et al., 2006), although variations in threshold values for landscape variables differ among regions (Rhodes et al., 2008). This suggests that occupancy in modeled high‐quality habitat may be lower than expected where the local landscape is fragmented.

The MaxEnt model clearly outperformed a site‐based habitat quality index calculated from browse tree availability and diversity at ground‐truth sites when predicting koala occupancy at those sites. This is consistent with the view that the determinants of koala habitat are likely to include a range of features including tree species, soil type, moisture, topography, elevation, and especially disturbance variables such as wildfire frequency (Lunney et al., 2007), all of which are accounted for by the model. In addition, there was considerable uncertainty in how to allocate tree species into different classes of browse quality (Appendix S1). More quantitative data on koala diet would be required to more reliably allocate tree species to different classes and to set appropriate weights in developing such a habitat quality index. This has implications for directing conservation actions or management mitigations for koalas. Identification of sites based solely on browse tree species is likely to be less accurate than habitat models that consider a suite of variables.

### 4.3 Model limitations

Some limitations in the MaxEnt model were evident. In particular, ground‐truthing identified that patches of rainforest, which do not contain browse species for koalas, may be overpredicted by the model at lower elevations. Prediction of rainforest as habitat was most apparent for smaller patches surrounded by otherwise suitable eucalypt forest. As an example, one ground‐truth site within a patch of rainforest contained emergent *Eucalyptus saligna,* a preferred browse species, and the patch itself was also in close proximity to eucalypt forest. Koalas were recorded calling at this site, but it is not known whether the calls originated inside or outside the patch of rainforest. Alternatively, while large patches of rainforest do not represent habitat for koalas, their fringes, as well as small patches, may be used for shelter, such as during hot weather.

### 4.4 Acoustic surveys and occupancy modeling

Another key result of field validation at ground‐truth sites was confirmation of the effectiveness of acoustic recorders at detecting male mating bellows, in conjunction with occupancy modeling. Acoustic surveys were much more effective than concurrent pellet searches (authors’ unpubl. data), probably because pellets are difficult to locate in some forest types (e.g., moist forests or where a dense understorey and litter is present). It is well known that koala pellet detectability depends on ground layer complexity and that pellet decay rates vary within and among vegetation communities, being notably faster in moist types (Cristescu, Goethals, Banks, Carrick, & Frère, 2012).

Koalas were recorded acoustically on 29% of ground‐truth sites (42% using all methods—acoustics, scats, and sightings; authors unpubl. data). This is a relatively high level of naïve occupancy given that a number of these sites were selected to test model performance in areas modeled as low suitability, indicating naïve occupancy in better quality habitat would be higher. This has implications for the potential of northeast NSW to support a previously overlooked, but large koala population. Previous surveys for koalas in northern NSW have recorded lower levels of detection than our survey. For example, a regional survey of northern NSW using playback and spot‐lighting recorded koalas at 12% of sites (Kavanagh et al., 1995). We suggest that acoustic recorders represent an innovative and efficient method for surveying and monitoring koalas and that the status of koalas in the northeast forests warrants re‐assessment.

## CONFLICT OF INTEREST

None declared.

## AUTHORS’ CONTRIBUTIONS

Bradley Law developed concept, designed project, led manuscript production, and collected field acoustic data. Gabriele Caccamo led spatial modeling and contributed to manuscript writing. Paul Roe and Anthony Truskinger provided acoustic expertise to extract koala calls from field recordings. Traecey Brassil involved in database management, provided logistical support, and contributed to writing. Leroy Gonsalves performed occupancy modeling and model validation using field data and contributed to writing. Anna McConville performed validation of computer matches with koala calls and field assistance. Matthew Stanton contributed to the collection of browse tree and scat data at ground‐truth sites.
