# Supplementary material for: Development and field validation of a regional, management‐scale habitat model: A koala Phascolarctos cinereus case study
Source: Ecol Evol. 2017 Aug 11;7(18):7475–89. doi: 10.1002/ece3.3300 (PMC5606888; doi:10.1002/ece3.3300)
Supplement: Supplementary file 1 [file ECE3-7-7475-s001.docx]

**Development and field validation of a regional, management-scale habitat model: a koala *Phascolarctos cinereus* case study**

**Supporting Information**

*Calculating the Site Habitat Quality Index*

First, species of Koala browse trees were classified into five classes of varying quality, based on literature reporting primary, secondary and supplementary browse species, and expert opinion (Table S1). Class 1 and Class 2 species generally refer to those browse species that represent high food quality for Koalas and would represent high quality breeding habitat. Class 3 represents species of lower quality, although they are still likely to support some Koala breeding habitat and low density populations. Class 4 species are likely to represent marginal habitat, but may be important for Koala movement and dispersal (McAlpine *et al.* 2006). Species rarely used by Koalas were Class 5.

In addition to browse tree basal area, browse tree diversity is also likely to contribute to habitat quality at a site, because different browse species could provide different nutrients and a more diverse stand of browse trees is likely to provide a more resilient food base at times of habitat disturbance or stress (e.g. drought) (Smith 2004). Browse tree diversity was calculated using the Shannon-Wiener Index for counts of tree species in Class 1, 2 and 3. Class 4 was omitted from diversity calculations as they were considered to have lower value.

Browse tree basal area and diversity were combined into a site scale index of habitat quality using the following formula:

**Site Habitat Quality** = *[(0.8*basal area of Class 1 trees) + (0.6*basal area of Class 2 trees) + (0.25*basal area of Class 3 trees) + (0.1*basal area of Class 4 species) + (0*basal area of all other trees)] ^0.6^ * [site Class 1, 2 & 3 browse tree diversity] ^0.4^*

We used strike rates of use for different browse trees derived from pellet counts beneath trees across a range of local studies as a guide to select weights for browse tree classes. For example, *E. tereticornis* is a Class 1 species and it has strike rates of 0.7 (the overall proportion of trees sampled of a given species that had koala faecal pellets) at Noosa, Queensland (Callaghan *et al.* 2011). We weighted browse tree availability higher than browse tree diversity because Koala habitat quality can still be high when browse diversity is low. We also experimented with alternative weightings and settled on values that yielded a higher r^2^ to optimise the relationship prior to comparing against the MaxEnt model output.

Table S1: Classification of tree species into 5 different browse qualities for Koalas based on literature and expert opinion (as an example see NSW Koala Recovery Plan 2007).

| **Class 1** | **Class 2** | **Class 3** | **Class 4** | **Class 5** |
| --- | --- | --- | --- | --- |
| *E. acaciiformis* | *E. biturbinata* | *E. globoidea* | *C. gummifera* | all other |
| *E. microcorys* | *E. canaliculata* | *A. torulosa* | *C. intermedia* |  |
| *E. robusta* | *E. glaucina* | *E. agglomerata* | *E. acmenoides* |  |
| *E. tereticornis* | *E. largeana* | *E. cameronii* | *E. pilularis* |  |
| *E. viminalis* | *E. moluccana* | *E. eugeniodes* | Ironbark spp. |  |
| *E. amplifolia* | *E. propinqua* | *E. grandis* | *M. quinquenervia* |  |
|  | *E. punctata* | *E. laevopinea* | Stringybark spp. |  |
|  | *E. radiata* | *E. nobilis* | *E. williamsiana* |  |
|  | *E. seeana* | *E. obliqua* |  |  |
|  |  | *E. quadrangulata* |  |  |
|  |  | *E. resinifera* |  |  |
|  |  | *E. rummeryi* |  |  |
|  |  | *E. saligna* |  |  |
|  |  | *E. siderophloia* |  |  |
|  |  | *E. signata* |  |  |
|  |  | *E. tindaliae* |  |  |
|  |  | *E. caliginosa* |  |  |
